# Assessing Apps for Patients with Genitourinary Tumors Using the Mobile Application Rating Scale (MARS): Systematic Search in App Stores and Content Analysis

**DOI:** 10.2196/17609

**Published:** 2020-07-23

**Authors:** Miguel Ángel Amor-García, Roberto Collado-Borrell, Vicente Escudero-Vilaplana, Alejandra Melgarejo-Ortuño, Ana Herranz-Alonso, José Ángel Arranz Arija, María Sanjurjo-Sáez

**Affiliations:** 1 Hospital General Universitario Gregorio Marañón Instituto de Investigación Sanitaria Gregorio Marañón Madrid Spain

**Keywords:** genitourinary cancer, mobile apps, eHealth, mHealth, rating tool

## Abstract

**Background:**

The large number of available cancer apps and their impact on the population necessitates a transparent, objective, and comprehensive evaluation by app experts, health care professionals, and users. To date, there have been no analyses or classifications of apps for patients with genitourinary cancers, which are among the most prevalent types of cancer.

**Objective:**

The objective of our study was to analyze the quality of apps for patients diagnosed with genitourinary cancers using the Mobile Application Rating Scale (MARS) and identify high-quality apps.

**Methods:**

We performed an observational cross-sectional descriptive study of all smartphone apps for patients diagnosed with genitourinary cancers available on iOS and Android platforms. In July 2019, we searched for all available apps for patients with genitourinary cancers (bladder, prostate, cervical, uterine, endometrial, kidney, testicular, and vulvar) or their caregivers. Apps were downloaded and evaluated, and the general characteristics were entered into a database. The evaluation was performed by 2 independent researchers using the MARS questionnaire, which rates 23 evaluation criteria clustered in 5 domains (Engagement, Functionality, Esthetics, Information, and Subjective Quality) on a scale from 1 to 5.

**Results:**

In total, 46 apps were analyzed. Of these, 31 (67%) were available on Android, 6 (13%) on iOS, and 9 (20%) on both platforms. The apps were free in 89% of cases (41/46), and 61% (28/46) had been updated in the previous year. The apps were intended for prostate cancer in 30% of cases (14/46) and cervical cancer in 17% (8/46). The apps were mainly informative (63%, 29/46), preventive (24%, 11/46), and diagnostic (13%, 6/46). Only 7/46 apps (15%) were developed by health care organizations. The mean MARS score for the overall quality of the 46 apps was 2.98 (SD 0.77), with a maximum of 4.63 and a minimum of 1.95. Functionality scores were quite similar for most of the apps, with the greatest differences in Engagement and Esthetics, which showed acceptable scores in one-third of the apps. The 5 apps with the highest MARS score were the following: “Bladder cancer manager,” “Kidney cancer manager,” “My prostate cancer manager,” “Target Ovarian Cancer Symptoms Diary,” and “My Cancer Coach.” We observed statistically significant differences in the MARS score between the operating systems and the developer types (*P*<.001 and *P*=.01, respectively), but not for cost (*P*=.62).

**Conclusions:**

MARS is a helpful methodology to decide which apps can be prescribed to patients and to identify which features should be addressed to improve these tools. Most of the apps designed for patients with genitourinary cancers only try to provide data about the disease, without coherent interactivity. The participation of health professionals in the development of these apps is low; nevertheless, we observed that both the participation of health professionals and regular updates were correlated with quality.

## Introduction

Genitourinary cancers represent 25% of all types of cancer [1]. For men, the most commonly diagnosed cancers are prostate cancer and bladder cancer, while for women, cervical cancer and uterine cancer are the most common [2]. Although these cancers are leading causes of death, recent advances in diagnosis and therapy have led to significant improvements in the overall survival of these patients [1].

The chronification of many genitourinary cancers and the special features of newer treatments, such as oral anticancer agents and immunotherapy, have also changed the profile of patients with this type of cancer [3-5]. As a result of the more exhaustive amount of information that patients and relatives need to know about the characteristics of the disease and treatment, there are new challenges for improving communication between patients and caregivers, and greater opportunities for contact with health care professionals. In this regard, information and communications technologies, especially mobile apps and remote assistance services [6-8], could help to improve the autonomy and communication options of these patients [9].

Currently, more than 200 health apps are released daily, and in the last 2 years, the number of available apps has doubled to reach more than 300,000 [10]. This development, which has not been specifically regulated, has led to the diffusion of some poor-quality apps [11,12]. Apps are downloaded from one of the operating system stores (“Play Store” for Android and “App Store” for iOS), where they are valued based on only 2 criteria (ie, the number of downloads and the user ratings) [13,14]. They are nonspecific search engines that do not enable the user to apply filters to assess their disease, the purpose of the app, or the quality of the app [6]. Consequently, searching for high-quality information is becoming even more difficult, with the result that the user downloads apps of uncertain reliability that are likely not the most appropriate option for his/her needs [15-17]. This aspect is particularly important for patients with cancer, where receiving poor-quality information may have a negative impact on prognosis [18].

The large number of available health care apps and their impact on the population necessitates a transparent, objective, and comprehensive evaluation by app experts, health care professionals, and users [15,19,20]. There are several methods to evaluate the quality of health apps. The most appropriate for use in online stores where patients can search and contrast health care apps is the Mobile Application Rating Scale (MARS). This tool provides a simple, quantitative, and validated system that enables rapid evaluation with little variation [12,13,21]. The number of apps for patients with cancer is continuously increasing owing to the availability of new information, requirements for communication, and the empowerment of patients who wish to participate in their care [5,9]. However, there is no standardized methodology for the classification, assessment, and validation of apps for patients with genitourinary cancers, although MARS is the most widely recommended.

The objective of our study was to analyze the quality of apps for patients diagnosed with genitourinary cancers using the MARS scale to identify high-quality apps.

## Methods

### Study Design

We performed an observational cross-sectional descriptive study of all smartphone apps for patients diagnosed with genitourinary cancers available on the iOS and Android platforms.

Our study followed a methodology to select the apps and adhered to the PRISMA (Preferred Reporting Items for Systematic review and Meta-Analysis Protocols) guidelines [22]. In July 2019, a search was conducted in the App Store (iOS) and Play Store (Android) within the categories “medicine” and “health and fitness.” The terms used in this search were the following: “bladder cancer,” “kidney cancer,” “testicular cancer,” and “prostate cancer” for urological tumors and “reproductive cancer,” “endometrial cancer,” “cervical cancer,” “uterine cancer,” “ovarian cancer,” and “vulvar cancer” for gynecological tumors.

Once the search was completed, all available information on the platform was analyzed, and only apps that were in English or Spanish and intended for patients and caregivers were selected. For this study, we excluded apps aimed specifically at health care professionals, those for charitable purposes or without scientific content, and those not specific to genitourinary cancers. Apps that met the indicated criteria were downloaded and evaluated, regardless of cost. The iOS apps were downloaded to an iPhone 8 (version 12.3.2) and the Android apps were downloaded to a Xiaomi Mi A1 (version 9.0).

### Characteristics and Content of the Apps

The general characteristics of the applications were entered into a database. Recorded characteristics included the name, platform (Android or iOS), cost (€), category (medicine and health and fitness), date of the last update, language, and target type of cancer. The content of the applications was classified into 1 of 3 categories according to its purpose: informative, preventive, and diagnostic. Furthermore, any information about the participation of health professionals in the app design or development was included. Qualified professionals were considered to have contributed to the app contents if the app had been developed by health care organizations such as local health authorities, universities, scientific societies and foundations, and hospitals.

### MARS Evaluation

The quality of the apps was then assessed using MARS. This methodology includes 23 evaluation criteria, clustered within 5 domains: (1) “Engagement,” which assesses the entertainment, customization, and interactivity of the app (feedback, reminders, and notifications); (2) “Functionality,” which examines the functionality of the app, ease of use, transition between screens, and intuitive design; (3) “Esthetics,” which assesses graphic design, visual appeal, and stylistic consistency; (4) “Information,” which evaluates the quality of the content (text, measures, and references), determined by the credibility of the source; and (5) “Subjective quality,” which determines whether the app could be recommended to people who might benefit from it, if they would be prepared to pay for it, how many times it would be used, and what overall star rating it would be given. Each evaluation criterion was rated from 1 to 5 (1=Inadequate, 2=Poor, 3=Acceptable, 4=Good, 5=Excellent) [12,13,22]. The MARS evaluation was carried out by 2 independent researchers with experience in app design and development and familiarity with genitourinary cancers. After all the evaluation criteria were scored, the mean score of the domains was calculated to obtain the total mean MARS score, which describes the overall quality of the app.

The quantitative variables were described using mean and standard deviation. The categorical variables were described using frequencies and percentages. The numerical variables were compared using the *t* test. A weighted Cohen κ test was performed to guarantee the reliability of those data analyzed by 2 independent observers, and the joint probability agreement (estimated as the percentage of times the raters agreed on an item) was measured. Results with a *P* value <.05 were considered statistically significant. Data were analyzed using Stata (version IC-15, StataCorp LLC).

## Results

### Study Design

**Figure 1:**
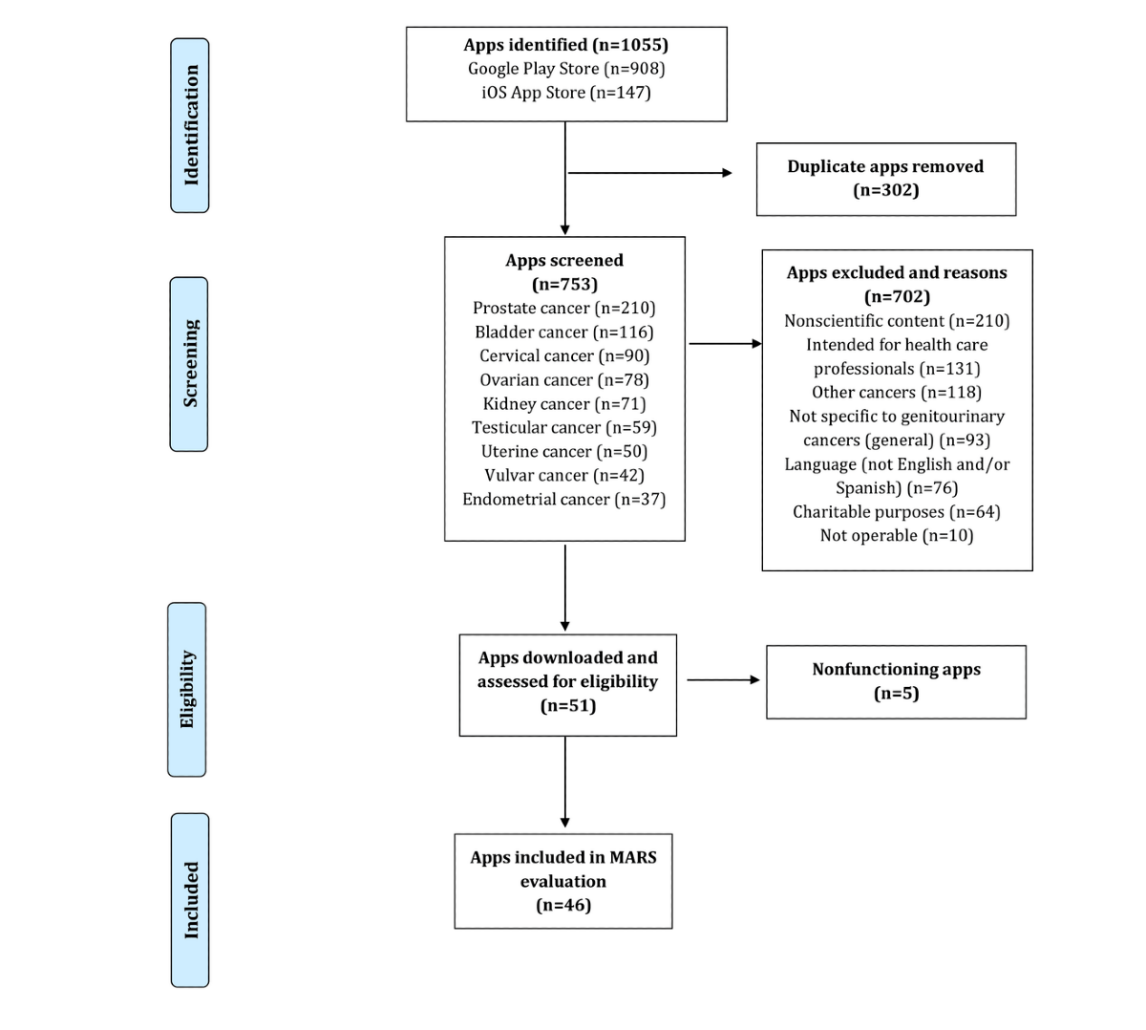
Study flowchart. MARS: Mobile Application Rating Scale.

The app search provided a total of 1055 apps, of which 51 were finally downloaded (Figure 1).

### Characteristics and Content of the Apps

At the time of the MARS evaluation, 5 of those 51 applications had been removed from the store or could not be opened, leading to a final total of 46. Of these, 31 (67%) were available on Android, 6 (13%) on iOS, and 9 (20%) on both platforms. Most of the apps (89%, 41/46) were free, with only 5 apps (11%, 5/46) requiring payment (mean cost of €3.51 [US $3.98], SD 1.21). The general characteristics of the apps are shown in Table 1. Table 2 describes the characteristics of the apps.

Most of the apps were informative (63%, 29/46), followed by preventive (24%, 11/46) and diagnostic (13%, 6/46). Only 7 of the 46 apps (15%) were developed by health care organizations, which included 3 scientific societies (43%), 3 universities (43%), and 1 hospital (14%). Regarding the type of cancer, 27/46 (59%) were intended for patients with urological cancers and 21/46 (47%) for those with gynecologic cancers. Apps for urological cancers were intended for prostate cancer in 14/46 apps (30%), testicular cancer in 7/46 (15%), bladder cancer in 4/46 (9%), and kidney cancer in 2/46 (4%). Gynecologic cancers were represented by cervical cancer in 9/46 apps (20%), ovarian cancer in 8/46 (15%), uterine cancer in 2/46 (4%), endometrial cancer in 2/46 (4%), and vulvar cancer in 1/46 (2%). One app contained information on cervical, testicular, and ovarian cancer.

**Table 1:** General characteristics of the apps.

Characteristics		Apps, n (%)
**Platform**		
	Android	31 (67)
	Android/iOS	9 (20)
	iOS	6 (13)
**Cost**		
	No	41 (89)
	Yes	5 (11)
**Category**		
	Medicine	26 (57)
	Health and fitness	20 (43)
**Year of the last update**		
	2014	1 (2)
	2015	3 (7)
	2016	2 (4)
	2017	12 (26)
	2018	28 (61)
**Language**		
	English	43 (94)
	Spanish	2 (4)
	English/Spanish	1 (2)

**Table 2:** Characteristics of the apps analyzed^a^.

Name of the app	Type of cancer	Purpose^b^			Platform		Free	Updated in the last year	Developed by a health organization	Language^c^	
		I	P	D	iOS	Android				E	S
Ball Checker	Testicular			✓	✓	✓	✓	✓	✓		✓
Best Prostate Cancer Treatment	Prostate	✓			✓		✓	✓		✓	
Bladder cancer (Bedieman)	Bladder	✓				✓	✓	✓		✓	
Bladder cancer manager	Bladder		✓		✓		✓	✓		✓	
Cancer cervix fact	Cervical	✓				✓	✓	✓		✓	
Cáncer de cuello uterino	Cervical	✓				✓	✓			✓	
Cáncer de ovarios	Ovarian	✓				✓	✓			✓	
Cancer de prostata (Anastore)	Prostate	✓				✓	✓			✓	
Cancer de prostata (Pen Drouzi)	Prostate	✓				✓	✓	✓		✓	
Cancer de RIÑON	Kidney	✓				✓	✓			✓	
Cáncer de vejiga	Bladder	✓				✓	✓			✓	
Cáncer testicular (Anass apps)	Testicular	✓				✓	✓			✓	
Cancer testicular (Expert Health Studio)	Testicular	✓				✓	✓	✓		✓	
Cancer testicular (Health Advice Ideas)	Testicular	✓				✓	✓	✓		✓	
Cancer testicular (Pen Drouzi)	Testicular	✓				✓	✓	✓		✓	
Cáncer uterino	Uterine	✓				✓	✓			✓	
Cervical cancer (Bedieman)	Cervical	✓				✓	✓	✓		✓	
Cervical cancer (Natural health care)	Cervical	✓				✓	✓	✓		✓	
Cervical cancer (Nougat spring)	Cervical	✓				✓	✓	✓		✓	
Cervical cancer (Personal Remedies LLC)	Cervical		✓		✓	✓		✓		✓	
Common causes of cervical cancer	Cervical		✓			✓	✓			✓	
El cancer de vulva	Vulvar	✓				✓	✓			✓	
Endometrial cancer (Bedieman)	Endometrial	✓				✓	✓	✓		✓	
Endometrial cancer (online Global Groups)	Endometrial	✓				✓	✓	✓		✓	
Global Pap App	Cervical		✓		✓		✓	✓		✓	
How to prevent ovarian cancer	Ovarian	✓				✓	✓	✓		✓	
IPCRC (Prostate Ca Calculator)	Prostate			✓		✓	✓			✓	
itsaMANTHING-Prostate cancer	Prostate	✓			✓	✓	✓		✓	✓	
Kidney cancer manager	Kidney		✓		✓		✓	✓		✓	
My Cancer Coach	Prostate	✓				✓	✓	✓	✓	✓	
My prostate cancer manager	Prostate		✓		✓		✓	✓		✓	
OddBalls-Check Yourself	Testicular		✓			✓	✓			✓	
Ovarian cancer (Personal Remedies LLC)	Ovarian		✓		✓	✓		✓		✓	
Ovarian Cancer Awareness	Ovarian	✓				✓	✓			✓	
Ovarian cancer symptoms diary	Ovarian		✓		✓	✓	✓		✓	✓	
Prostate cancer (Dinatale)	Prostate	✓				✓	✓	✓		✓	
Prostate cancer (Personal Remedies LLC)	Prostate		✓		✓	✓		✓		✓	
Prostate Cancer Calculator	Prostate			✓		✓	✓			✓	
Prostate PRO-Tracker	Prostate			✓	✓		✓		✓	✓	
ProstateCheck	Prostate			✓	✓	✓			✓		✓
Reproductive cancers	Ovarian, cervical, testicular	✓				✓	✓	✓		✓	
Rotterdam Prostate Cancer Risk	Prostate			✓	✓	✓		✓	✓	✓	✓
Target Ovarian Cancer Symptoms Diary	Ovarian		✓		✓	✓	✓	✓		✓	
Treat prostate cancer	Prostate	✓				✓	✓			✓	
Treating bladder cancer	Bladder	✓				✓	✓	✓		✓	
Ways to treat uterine cancer	Uterine	✓				✓	✓	✓		✓	

^a^In apps with the same name, the developer is indicated in parentheses.

^b^I: informative; P: preventive; D: diagnostic.

^c^E: English; S: Spanish.

### MARS Evaluation

The mean MARS score for the overall quality of the 46 apps was 2.98 (SD 0.77), with a maximum of 4.63 and a minimum of 1.95 (Table 3).

The Functionality scores were similar for most of the apps. The apps provided adequate and rapid movement between the screens and menus. The greatest differences were found in the Engagement domain because of deficiencies in areas such as customization and interactivity. Similar differences were found in the Information domain because of the visual explanation and evidence base. The Esthetics domain showed acceptable scores in one-third of the apps; this was associated with a greater update rate and visual appeal.

**Table 3:** Mobile Application Rating Scale scores of the evaluated apps out of 5^a^.

Name of app	Engagement	Functionality	Esthetics	Information	Subjective quality	Overall
Bladder cancer manager	4.60	4.63	4.83	4.57	4.50	4.63
Kidney cancer manager	4.60	4.63	4.83	4.57	4.50	4.63
My prostate cancer manager	4.60	4.63	4.83	4.57	4.50	4.63
Target Ovarian Cancer Symptoms Diary	4.10	4.75	4.83	3.93	3.63	4.25
My Cancer Coach	4.10	4.63	3.67	4.50	4.25	4.23
Rotterdam Prostate Cancer Risk	3.20	4.63	3.67	4.43	3.25	3.83
ProstateCheck	3.40	4.88	3.50	3.36	3.25	3.68
Ball Checker	3.50	4.25	3.67	4.21	2.75	3.68
Prostate PRO-Tracker	3.40	4.75	3.67	4.21	2.88	3.64
Global Pap App	2.90	4.88	3.67	3.71	3.00	3.63
OddBalls - Check yourself	3.30	3.88	3.50	4.00	3.25	3.59
Prostate Cancer Calculator	3.80	4.63	2.00	3.71	3.5	3.53
itsaMANTHING-Prostate Cancer	2.60	4.88	3.17	3.71	3.13	3.50
Endometrial cancer (online Global Groups)	2.60	4.63	3.50	3.29	3.25	3.45
Prostate cancer (Dinatale)	2.50	4.63	3.67	3.64	2.38	3.36
IPCRC (Prostate Ca Calculator)	2.50	4.88	3.50	2.71	3.00	3.32
Treating bladder cancer	2.50	4.50	3.67	3.29	2.63	3.32
Ovarian Cancer Symptoms Diary	2.90	4.38	3.17	3.14	2.88	3.29
Cáncer de prostata (Pen Drouzi)	1.90	4.50	3.50	3.36	2.25	3.10
Cáncer testicular (Pen Drouzi)	1.90	4.50	3.50	3.36	3.25	3.10
Cervical cancer (Nougat spring)	2.20	4.75	2.50	2.93	2.75	3.03
Best Prostate Cancer Treatment	2.40	4.00	3.33	3.21	2.13	3.02
Bladder cancer	2.10	4.63	3.00	2.86	2.38	2.99
Cervical cancer (Personal Remedies LLC)	3.20	2.25	3.50	2.86	1.88	2.74
Ovarian cancer (Personal Remedies LLC)	3.20	2.25	3.50	2.86	1.88	2.74
Prostate cancer (Personal Remedies LLC)	3.20	2.25	3.50	2.86	1.88	2.74
Cancer testicular (Expert Health Studio)	1.80	3.50	3.33	3.14	1.63	2.68
Cancer testicular (Health Advice Ideas)	1.80	3.38	3.33	3.14	1.50	2.63
Reproductive cancers	1.60	4.50	3.00	2.43	1.50	2.61
Cervical cancer (Natural health care)	1.70	4.38	2.33	2.86	1.75	2.60
Cervical cancer (Bedieman)	1.40	3.75	2.50	2.29	1.75	2.34
Endometrial cancer (Bedieman)	1.40	3.75	2.50	2.29	1.75	2.34
How to prevent ovarian cancer	1.70	3.88	2.33	2.57	1.13	2.32
Ovarian Cancer Awareness	1.80	4.38	2.00	1.93	1.50	2.32
Treat prostate cancer	1.60	4.00	1.67	2.57	1.75	2.32
Cancer de próstata (Anastore)	1.60	3.50	2.17	2.71	1.50	2.32
Cancer de RIÑON	1.70	3.00	1.67	2.64	1.63	2.13
Cáncer de vejiga	1.70	3.00	1.67	2.64	1.63	2.13
Cancer testicular (Anass apps)	1.70	3.00	1.67	2.64	1.63	2.13
Cancer de cuello uterino	1.70	3.00	1.67	2.64	1.63	2.13
Cáncer de ovarios	1.70	3.00	2.00	2.57	1.25	2.10
Cáncer uterino	1.70	3.00	1.67	2.64	1.50	2.10
El cáncer de vulva	1.70	3.00	1.67	2.64	1.50	2.10
Common causes of cervical cancer	1.80	3.13	1.50	2.50	1.50	2.09
Ways to treat uterine cancer	1.60	3.50	1.33	2.00	1.63	2.01
Cancer cervix fact	1.40	3.63	1.33	2.14	1.25	1.95

^a^For apps with the same name, the developer is indicated in parentheses.

Comparison by the operating system (iOS and Android) revealed an overall MARS score of 3.64 for apps available in the App Store (n=15) and 2.19 for those available in the Play Store (n=40); the difference was statistically significant (*P*<.001). However, when the overall MARS scores were analyzed considering whether the apps were free (n=41) or required payment (n=5), the only significant differences were in the Functionality domain. Comparison by developer type revealed statistically significant differences between the apps that had been supported by a health organization (n=7), with a score of 3.70, and those that had not (n=39), with a score of 2.85 (Table 4).

Interrater agreement was substantial across all evaluation criteria, except for the Entertainment and Interest criteria of the Engagement domain and the Subjective Quality criterion “What is your overall star rating for the app?” The joint probability of agreement was >85% in all the items and >90% in 11 of the 23 evaluation criteria and 4 of the 5 domains analyzed (Table 5). The mean κ score for the 5 domains was 0.748, indicating that substantial agreement was observed between the 2 evaluators.

**Table 4:** Results of the Mobile Application Rating Scale evaluation: comparison by different characteristics.

Category	Operating system				Developer				Cost		
	Android (n=40)	iOS (n=15)	*P* value	Non–health organization (n=39)		Health organization (n=7)	*P* value	Free (n=41)		Paid (n=5)	*P* value
Engagement	2.30	3.45	<.001	2.34		3.30	.01	2.40		3.24	.06
Functionality	3.87	4.13	.32	3.85		4.63	.02	4.05		3.25	.03
Esthetics	2.75	3.84	<.001	2.83		3.50	.10	2.86		3.53	.16
Information	3.00	3.72	<.001	3.00		3.88	.002	3.12		3.27	.67
Subjective quality	2.18	3.05	.001	2.21		3.18	.01	2.35		2.43	.88
Overall	2.82	3.64	<.001	2.85		3.70	.01	2.96		3.14	.62

**Table 5:** Interrater agreement for the Mobile Application Rating Scale domains and evaluation criteria.

Domains and evaluation criteria		Weighted Cohen κ	Agreement (%)
**Engagement**		0.76	92.2
	Entertainment	0.55	89.4
	Interest	0.56	87.8
	Customization	0.86	96.8
	Interactivity	0.77	91.5
	Target group	0.64	88.8
**Functionality**		0.71	90.0
	Performance	0.68	89.9
	Ease of use	0.62	89.4
	Navigation	0.69	87.9
	Gestural design	0.64	87.9
**Esthetics**		0.80	93.6
	Layout	0.75	90.8
	Graphics	0.76	93.1
	Visual appeal	0.91	96.8
**Information**		0.79	93.6
	Accuracy of the app in the description (App/Play Store)	0.63	89.4
	Goals	0.72	93.6
	Quality of information	0.67	88.7
	Quantity of information	0.65	87.2
	Visual information	0.86	94.1
	Evidence base	0.66	94.1
	Credibility	0.61	89.4
**Subjective quality**		0.68	89.9
	Would you recommend this app to people who might benefit from it?	0.64	90.4
	Would you pay for this app?	0.74	90.8
	How many times do you think you would use this app in the next 12 months if it was relevant to you?	0.72	90.4
	What is your overall star rating of the app?	0.48	86.7

## Discussion

### Principal Findings and Comparison With Previous Work

Based on a systematic and validated questionnaire (MARS), our study provided an objective ranking of 46 apps for patients with genitourinary cancers available in the Apple and Android stores. Apps for patients with prostate and cervical cancer accounted for almost half of all the apps evaluated (30% and 17%, respectively). This frequency is consistent with the findings of the Globocan 2018 report [2], according to which genitourinary cancers have the highest global incidence (second in men and fourth in women). Therefore, the number of apps analyzed correlated with the incidence of the specific genitourinary cancers, unlike other types of cancer, such as lung and colorectal cancer, which are extremely prevalent, yet for which few apps have been released [15].

More than half of the apps (61%) had been updated in the last year and therefore provided better quality information, which is increasingly necessary given the advantages of apps in diagnosis and therapy in this area. Our result is comparable to that obtained in a review of 166 apps for patients with cancer, where it was observed that 52.4% had been updated in the previous year [15]. However, the proportion of apps that had been developed or promoted by health care organizations was very low (15.2%), thus potentially reducing the quality and reliability of the apps. This result was consistent with the conclusions of authors such as Giunti et al [14], who showed an evident absence of health professionals in the development of health care apps. Apps are mostly developed by non–health professionals who are creative and skilled in design but lack scientific knowledge.

Most of the apps included in our study were informative, with generic data on pathophysiology, treatments, and symptoms of individual cancers, as reported elsewhere [14]. An important component of some of these apps, such as Treating Bladder Cancer, was including information about the most common symptoms and signs of bladder cancer (hematuria, pain or burning sensation, and increased frequency in urination) that could alert patients to talk to the doctor. These features are important to reduce the risk of having a more advanced stage of cancer. We found that most of the informative apps, like those for cervical cancer, included information about treatment and prognosis. Nonetheless, this information should be given by physicians, and informative apps provide doctors with a tool to improve early diagnosis through successful screening. Additionally, as indicated by Bender et al [23], most of the apps try to increase awareness of cancer in the population. Very few apps focused on how to handle the disease after a diagnosis, correct administration of the treatment prescribed (eg, dosing, management of adverse effects, possible interactions with other long-term medications), and adequate monitoring of symptoms. Only 6 diagnostic apps (13.0%) were evaluated; of these, 5 were intended for patients with prostate cancer and 1 for patients with testicular cancer. Diagnostic apps are more frequent in other types of cancer, such as melanoma and breast cancer [16].

Several methods for the evaluation of mHealth apps have been developed, although in most cases, the absence of a systematic methodology and the fact that they were not developed by scientific professionals made their routine use impossible [12]. The MARS methodology, as reported by Stoyanov et al [21], is an easy-to-use, simple and logical tool that is considered highly reliable because it is promoted by expert technicians and health care professionals. This evaluation proposes a multidisciplinary analysis designed for all health care apps, with 5 domains covering the main aspects for correct evaluation. Stoyanov et al [13] reported that the MARS questionnaire showed high levels of internal consistency (Cronbach α=.9) and interrater reliability (two-way mixed intraclass correlation coefficient, 0.79; 95% CI, 0.75-0.83) when it was applied to rate 50 mental health apps.

As for the MARS score, our study showed a mean score of 2.98 for the overall quality of the apps, with a score of 3.13 for quality content. These scores were significantly higher than those found by Böhme et al [6] for mobile cancer apps for prostate cancer, breast cancer, and colorectal cancer (1.96). On the other hand, our results were similar to the scores of apps for other diseases. For example, the mean score found by Richardson et al [19] for mobile apps targeted to parents of infants in the neonatal intensive care unit was 3.37, without considering the subjective quality. Salazar et al [24] showed a mean score of 3.92 for mobile apps for the management of pain, while Siddique et al [25] found a median score of 3.70 for apps targeted to the care management of chronic kidney and end-stage renal disease. To note, 22 apps (48%) scored a value equal to or greater than 3 points (ie, “Acceptable”). Only 5 apps (11%) exceeded 4 points in the overall quality score (ie, “Good”). Jupp et al [3] found that the apps evaluated stood out in the Functionality domain, with high scores in most of them because they were developed to be highly efficient and easy to use. Consistent with our findings, these authors found that the 3 strategies necessary for optimal use of the smartphone among patients with cancer were the management of symptoms and medications, quality of the information resources, and ability to export data. In contrast, the Esthetics domain had a better correlation with the overall MARS score. It is assumed that this is due to the attempt to develop a more engaging appearance which is directly related to other features, such as a higher frequency of use.

In reports based on MARS for assessment of apps aimed at patients with cancer or other diseases, the domain that scored the lowest was Engagement. The main reason is that the apps were unable to make patients feel that they were participating in the management of their disease. We drew the same conclusion, with Engagement being the domain with the poorest mean score compared to the others. According to the literature, the fundamental aspects that can be improved in this section are customization, user interactivity, and entertainment, which leads to a higher score [3,20]. The participation of patients in the development, design, and validation of apps through focus groups considerably improves the score for this domain. Furthermore, the participation of patients in the management of their symptoms by registering and sending messages and reminders significantly enhances the health outcomes [26]. Collado et al [18] found that more than 40% of patients would be interested in communicating with their physician or pharmacist using an app.

The apps that scored best in the MARS evaluation were “Bladder cancer manager,“ ”Kidney cancer manager,“ and ”My prostate cancer manager.“ These apps were available in the App Store and stood out because of their high scores in the Engagement and Esthetics domains, as did the next 2 apps in the ranking, “Target Ovarian Cancer Symptoms Diary” and “My Cancer Coach.” The “Top 5” apps contained reminders and schedules and offered the possibility of registering analytical information and treatments prescribed, thus enabling a greater score in the Subjective Quality domain because they achieve the main goal. Likewise, the 4 best apps had an explicit preventive purpose, in contrast to the informative purpose that was more frequent in the global analysis.

Of note, 9 of the 10 best apps had been updated during 2018, and 5 of the 10 best apps had been developed by a health care organization. This is an important observation because neither of these 2 aspects is specifically evaluated in MARS. The analysis by the domain of the apps developed by health care organizations and those that were not revealed statistically significant differences for each of the items evaluated. Therefore, the quality of health apps is based on the frequency of updating and the participation of health care organizations in their development, thus confirming the hypothesis proposed elsewhere [15,27]. The first three apps (“Bladder cancer manager,” “Kidney cancer manager.” and “My prostate cancer manager”) had the same developer (Point of Care) which is a platform of health apps intended to patients and clinicians. However, clinicians were not involved in their development. Also, “Target Ovarian Cancer Symptoms Diary” had a clinical advisory panel made up of many oncology specialists who answer patients´ questions but they were not involved in promoting the app. Otherwise, “My Cancer Coach” was developed by some health organizations and the coaches formed a multidisciplinary team (nurses, physicians…) to provide reliable information to patients. However, the lowest scores were obtained for apps that are merely informative and provide links to other web pages but not their information.

The analysis by operating system revealed a statistically significant difference that was more favorable to the apps from the App Store than those from Play Store, probably because verification requirements for publishing and application are stricter for iOS than for Android.

Apps are beginning to show a significant impact on users´ health. Because of that, regulatory authorities are responsible for evaluating these technologies to control their availability in the stores. In 2013 and regarding this issue, the Food and Drug Administration (FDA) published a guide containing recommendations to assess the quality of these apps. However, due to the rise of health apps, an objective, comprehensive and clear evaluation of apps is necessary. This evaluation should allow users and health care professionals (who recommend them to patients) which apps meet minimum standards of quality and safety in their content.

Patients are exposed to unreliable information related to their health so quality certifications are needed to identify those apps that offer the best content for users. For example, App Saludable is a free and open-access certification given to some apps which were developed using strict guidelines in Spain [28]. This is one of the first certifications in Europe that evaluate the quality and safety of health apps.

### Limitations

MARS is limited by its subjectivity [13,21]. However, the high interrater reliability obtained between the 2 evaluators, both of whom had experience in the development and validation of health apps and were familiar with genitourinary cancers, highlights the considerable coherence of our results.

### Conclusions

The quality of health apps should be evaluated using approaches such as MARS to decide which apps could be prescribed to patients and to identify which features should be addressed to improve these tools. Most of the apps designed for patients with genitourinary cancers only try to increase awareness and provide data about the disease, without ensuring coherent interactivity. Although the participation of health professionals in the development of these apps is low, we observed that their participation was associated with the app quality and the recency of updates. Greater scores in quality were observed in iOS apps, although no correlation between quality and price was found.

## Abbreviations

FDA: Food and Drug Administration
MARS: Mobile Application Rating Scale
